# A strain-independent method to induce progressive and lethal pneumococcal pneumonia in neutropenic mice

**DOI:** 10.1186/s12929-015-0124-4

**Published:** 2015-03-25

**Authors:** Andres F Zuluaga, Beatriz E Salazar, Maria Agudelo, Carlos A Rodriguez, Omar Vesga

**Affiliations:** GRIPE [Grupo Investigador de Problemas en Enfermedades infecciosas], Medellín, Colombia; Department of Pharmacology and Toxicology, Medellín, Colombia; Department of Microbiology and Parasitology, Medellín, Colombia; Department of Internal Medicine, Universidad de Antioquia, Calle 70 No. 52-21, Medellín, Colombia; Infectious Diseases Unit, Hospital Universitario San Vicente Fundación, Medellín, Colombia

**Keywords:** Animal model, Murine, Pneumonia, *Streptococcus pneumoniae*, Mucin

## Abstract

**Background:**

Experimental models of pneumonia with penicillin non-susceptible *Streptococcus pneumoniae* (PNSSP) are hard to reproduce because the majority of strains with clinical relevance (like serotypes 6B, 9 V and 19 F) have low murine virulence. By optimization of culture and inoculum conditions of PNSSP (using porcine mucin), our aim was to develop a suitable, reliable and reproducible pneumonia mouse model for anti-infective pharmacology research.

**Results:**

Seven PNSSP strains, including serotypes 6B, 9 V, 14 and 19 F were included. Strain INS-E611 displayed the highest murine virulence and was chosen to validate the lung model. Nose-instilled pneumococci grew between 2.1 and 2.5 log_10_ CFU/g of lung in 24 hours when an optimized culture of bacterial cells was used, but animals were all alive and recovered of infection after 36 h. In contrast, inoculum supplementation with mucin led to 100% mortality related to a successful lung infection confirmed by histopathology. These findings were reproduced with all seven PNSSP strains in neutropenic mice. Immunocompetent animals cleared all strains spontaneously.

**Conclusions:**

This pneumonia model produces a progressive and uniformly fatal lung infection with diverse serotypes of PNSSP independently of their intrinsic murine virulence.

## Background

*Streptococcus pneumoniae* is a leading cause of infection in young children, the elderly and debilitated patients. Its spectrum of disease ranges from otitis media and sinusitis to life-threatening infections such as pneumonia and meningitis that kill every year almost one million children younger than 5 year-old [1]. A fully susceptible microorganism in the past, *S. pneumoniae* treatment options are currently endangered by the emergence and dissemination of multiple drug resistance (MDR), especially to β-lactams, macrolides and quinolones [2]. Despite massive, highly effective vaccination, *S. pneumoniae* is still very able to cause serious disease through those serotypes not included in the diverse polyvalent vaccines [3,4].

Animal models of human lung infection are essential tools for the preclinical testing and optimization of new drugs and vaccines, dose regimens and antibiotic combinations [5,6]. In the case of penicillin non-susceptible *Streptococcus pneumoniae* (PNSSP), the majority of strains of clinical importance (like serotypes 6B, 9 V and 19 F) have low virulence for mice [7-9]; as a natural consequence of this experimental limitation, published models of murine pneumococcal pneumonia are difficult to reproduce [10,11]. To increase the probability of inducing pneumonia, the microorganism is delivered by unnatural routes like intratracheal injection, which is technically difficult, time-consuming, traumatic, and leads to asymmetric deposition of microorganisms with high variance [10]. A natural method of delivery, like the aerosolization of the inoculum, is a potential biologic hazard for the experimenter, requires expensive equipment, and does not lead to success with strains without murine virulence [12]. An important disadvantage noted in the literature is that bacterial growth is either not determined [13] or poor (<1 log_10_ CFU/g of lung tissue) [14], and the intra- and inter-experimental variance is usually high [12], minimizing its relevance [15]. In consequence, available PNSSP pneumonia models are characterized by their lack of reliability, a severe hindrance to assess the efficacy of antibiotics against the many serotypes of this important human pathogen [12,16].

Here, by optimization of culture and inoculum conditions of PNSSP, our aim was to develop a suitable, reliable and reproducible pneumonia mouse model for anti-infective pharmacology research. The initial data of this work were presented at the 45^th^ Interscience Conference on Antimicrobial Agents and Chemotherapy.

## Methods

### Bacterial strains, supplementation and optimization of in vitro conditions

We studied two clinical strains of penicillin-resistant (INS-E611, E674) *Streptococcus pneumoniae* and five penicillin-intermediate isolates (E676, E678, E683, E684 and ATCC 49619), all of them provided by the Colombian National Institute of Health (*Instituto Nacional de Salud*, Bogotá, Colombia). The minimal inhibitory and bactericidal concentrations of these strains to several drugs were previously published [17], including also the capsular serotypes 6B (INS-E611), 9V (INS-E683), 14 (INS-E674, INS-676, INS-678, INS-684) and 19 F (ATCC 49619). Microorganisms were stored at −70°C using skim milk media (Becton Dickinson & Co. Sparks, MD, USA). The standardization of the optimal culture conditions of pneumococci to produce ≥9 log_10_ CFU/mL of early log-phased cells without autolysis was described elsewhere [17]. Briefly, cells from frozen stock were recovered by two successive passages on solid media (5% sheep blood Trypticase soy agar supplemented with 0.5% yeast extract) incubated during 15 hours (h) under 5% CO_2_ atmosphere at 37°C. Then, 10 colonies from the second passage were diluted in 10 mL of Todd Hewitt Broth (THB, Becton Dickinson & Co, Sparks, MD, USA) supplemented with 2.5% horse blood and 2% yeast extract (adjusting pH to 7.8) and incubated during 12 h under a 5% CO_2_ atmosphere at 37°C. Finally, 1 mL of the bacterial suspension was diluted again in 9 mL of supplemented THB and incubated under the same atmosphere during 4 to 5 h (early-log phase) until obtaining an O.D_580nm_ of 0.8 that, according to previous standardization procedures, corresponds to a final inoculum of ~8 log_10_ cells per mL, ready for in vivo experiments.

### Animals

Six week old, murine pathogen free mice from the our strain Udea:ICR(CD-1) [18], weighing 23–27 g, were used in all experiments, including females for the pneumonia model and males for assays of pneumococcal virulence. All animals were given food and water *ad libitum* and the study was approved by the University of Antioquia Animal Care and Experimentation Ethics Committee. Mice were rendered neutropenic (<100 neutrophils/μL) by two intraperitoneal injections of cyclophosphamide (Cytoxan®, BMS, Princeton, NJ) 4 days (150 mg/kg) and 1 day (100 mg/kg) before infection [19].

### In vivo assays of pneumococcal virulence

Groups of at least 2 immunocompetent male mice were inoculated with each strain in both thighs with 0.1 mL of a bacterial suspension having ~8 log_10_ CFU/mL. After 26 h, mice were euthanized and thighs, lungs, liver, kidney and spleen were aseptically removed, homogenized and cultured on 5% sheep blood trypticase soy agar during 18 h under 5% CO_2_ at 37°C for bacterial counting. Five colonies of each strain were selected from the solid media to be used in up two additional cycles of the same animal model. To describe the clinical findings observed in infected animals during thigh passages, we designed the following score of murine virulence: 4+ (the mice died during the assay), 3+ (mice ended alive but with systemic illness), 2+ (mice with localized sickness) and 1+ (mice without clinical signs of disease). The objective of this step was to maximize the probability of success in our first attempts by selecting the penicillin non-susceptible strain with the highest virulence (i.e. a strain that induces lethality and dissemination to distant organs) for the development of the pneumonia model without the addition of porcine mucin. Subsequent experiments did not include thigh passages.

### Induction of pneumonia in mice

Before infection, female neutropenic mice were anesthetized by a subcutaneous injection (100 μL) of 100 mg/kg of ketamine (Ketalar®, Parke-Davis, Ecuador) plus 10 mg/kg of xylazine (Rompun®, Bayer S.A, Brazil), and the eyes were rubbed with Viscotears® (Dr. G. Mann Pharma) to prevent corneal ulcerations. Each mouse was inoculated by intranasal instillation with 50 μL of a bacterial suspension containing ~8 log_10_ CFU/mL. After instillation, animals were held in a vertical position during 10 min hanging from their incisor teeth to favor migration of bacteria to the alveoli by gravity. A minimum of three mice per experimental group were sacrificed in at least three of the following time-points: 0, 1, 6, 12, 18, 24, 32, 36, 38, 40 and 48 h post-infection (experiments were performed at least twice to test repeatability); in addition, a minimum of four animals were observed during 120 h (survival animals) to estimate the lethality. At the selected time points, we euthanized the mice by cervical dislocation, opened the thorax under aseptic technique to remove both lungs in block (cutting at the point of bronchial bifurcation from the trachea), homogenized it in 2.7 mL of sterile saline, and plated sequential 10-fold dilutions for colony counting (CFU/g). Both lungs weighed in average 0.3 g. Control animals, uninfected but anesthetized and instilled with 50 μL sterile normal saline, were used in all experiments. We checked the animals every 6 hours during the survival experiments and processed immediately any mouse found dead. Animals fulfilling any of these criteria were humanely euthanized: (a) inability to obtain feed or water, or (b) moribund state or no response to gentle stimuli.

The data, expressed as bacterial counts in the lungs of each animal (log_10_ CFU/g), were stored using Microsoft Excel 2013 (Microsoft Corp., Seattle, WA, USA) and analyzed and graphed in Prism 6.0 (GraphPad Software, San Diego, CA, USA).

### Development of the lethal and reproducible model of pneumonia

For each strain of *S. pneumoniae* (E611, E674, E676, E678, E683, E684, and ATCC 49619), two groups of neutropenic mice received the bacterial inoculum without or with mucin supplementation to test its impact on the lethality and reproducibility of the model. When supplemented, the bacterial inoculum was mixed with porcine mucin (M-1778; Sigma Chemical Company, St Louis, MO) just before nasal instillation. For this instance, a mucin stock solution (10% [wt/vol]) was diluted 1:1 with the pneumococcal suspension in supplemented THB with ~8 log_10_ CFU/mL prepared as describe above, for a final mucin concentration of 5%.

### Impact of neutrophils on the model

To assess the impact of neutrophils in the development of the model we infected groups of PNSSP immunocompetent mice with each one of the seven *S. pneumoniae* strains (E611, E674, E676, E678, E683, E684, and ATCC 49619) using the same conditions for inoculum preparation and mucin supplementation described above. Animals were sacrificed at 0, 14, 24, 38 and 120 hours for CFU count per gram of lung.

### Histopathology

We compared the morphological changes 38 h after mice infection fixing both lungs with 10% buffered formalin and staining with hematoxylin and eosin. After blind reading by a pathologist, samples of three different groups of mice were compared: (a) mice inoculated only with mucin (controls, not infected), (b) mice infected with *S. pneumoniae* INS-E611 without mucin, or (c) mice infected with the same strain but supplementing the inoculum with mucin.

### Statistical analysis

All data are presented as geometric mean ± SD. The net bacterial growth (G) was defined as the change in bacterial density calculated as the difference in mean log_10_ CFU/g at the zenith (highest bacterial growth) and nadir (lowest bacterial load in tissue) for the different strains. Differences at zenith on bacterial burden in lungs of mice infected with an optimized inoculum of pneumococci with or without mucin were analyzed by the Mann–Whitney test [20]. Data were considered significant when P values were <0.05 by the use of two-tailed significance levels.

## Results

### Strains and murine virulence

Table 1 summarizes the capsular serotypes, pattern of antibiotic susceptibility, and murine virulence of each strain studied in vivo without mucin enhancement. Despite the fact that all 7 strains were encapsulated with almost the same capsular densities ranging from 1.029 to 1.034 [21], murine virulence varied widely among them. Two out of 7 (29%) strains exhibited some degree of virulence (≥3+) after 48 h of infection and 5 of 7 (71%) induced minimal to no clinical changes (non-virulent strains) after several passages in thighs.

**Table 1:** **Capsular serotypes, antibiotic susceptibility patterns and in vivo virulence of the seven pneumococcal strains included in the study**

***S. pneumoniae*** **strain**	**Capsular serotype**	**Pattern of susceptibility to antibiotics**	Mouse virulence*
INS-E611	6B	DRSP (PEN-R, CRO-I, SXT-R)	4+
INS-E674	14	DRSP (PEN-R, CRO-I, SXT-R)	1+
INS-E676	14	DRSP (PEN-I, SXT-R)	1+
INS-E678	14	DRSP (PEN-I, SXT-R)	1+
INS-E683	9 V	DRSP (PEN-I, SXT-R)	2+
INS-E684	14	PNSSP (PEN-I)	2+
ATCC 49619	19 F	PNSSP (PEN-I)	3+

Abbreviations. INS: Instituto Nacional de Salud; CSF: Cerebro-Spinal fluid; DRSP: Drug-Resistant *S. pneumoniae;* PNSSP: Penicillin-Non-Susceptible *S. pneumoniae*; PEN: Penicillin; CRO: Ceftriaxone; SXT: Trimethoprim-sulfamethoxazole; CHL: Chloramphenicol; R: Resistant; I: Intermediate; *Mouse virulence of 4+ means that mice died during the assay, 3+: mice with systemic illness, 2+: mice with localized sickness and 1+: mice without clinical signs of disease.

Strain INS-E611 (PNSSP) exhibited the highest murine systemic virulence, killing immunocompetent mice early (<24 h after thigh infection) with dissemination to distant organs after passes in vivo (data not shown). According to these results, we selected INS-E611 as the non-susceptible strain to standardize the optimized pneumonia model.

### Repeatability of nasal route to induce pneumonia in neutropenic mice

**Figure 1:**
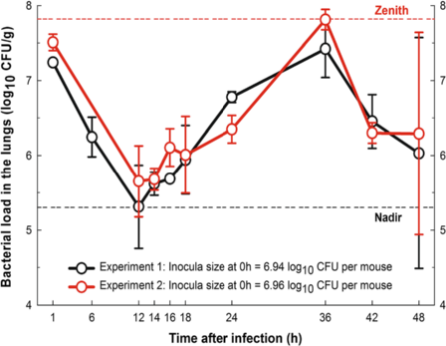
**Reproducibility of the pneumonia model using an optimized culture of** ***S. pneumoniae*** **.** In vivo growth dynamics of *S. pneumoniae* INS-E611 in neutropenic mice using an optimized inoculum. Data from two independent experiments; the circles represent the mean (three mice per time-point) and the error bars the standard deviation.

Figure 1 illustrates the pulmonary bacterial load at different times in two independent experiments after nasal instillation of *S. pneumoniae* INS-E611 (without mucin). In both experiments, we used a previously standardized methodology to produce a high quality culture that prevents bacterial autolysis [17]. The inoculum per mouse was 6.94 and 6.96 log_10_ CFU for experiments 1 and 2, respectively. The dynamics of in vivo bacterial growth were overlapped using the data from both experiments. The nadir was detected at 12 h (5.3 ± 0.56 and 5.65 ± 0.48 log_10_ CFU/g, for experiments 1 and 2, respectively), growth restarted at 14 h, and the zenith was reached 36 h after infection (7.42 ± 0.38 and 7.81 ± 0.14 log_10_ CFU/g, for experiments 1 and 2, respectively). The net growth during the 24 hours spanning from nadir to zenith (G_12→36h_) ranged from 2.11 to 2.16 log_10_ CFU/g. In spite of this bacterial growth, all animals recovered and were alive and healthy 120 h after infection; it correlated with a marked reduction (1 log) in the number of bacteria per gram of lung at 38 h (6.50 ± 0.43 log_10_ CFU/g) and an increment in the variance at 48 h (SD > 1.3 log_10_ CFU/g in both experiments), as expected when the infection is being cleared. Additionally, the bacterial burden in organs other than the lungs (i.e. blood and spleen) was also highly variable (SD ranging from 0.54 to 1.04 log_10_ CFU/g) with a mean load of only 2.48 log_10_ CFU/g, close to the limit of detection (2.0 log_10_ CFU/g). Therefore, the nasal instillation of a highly virulent strain of PNSSP was followed by significant and reproducible net growth in vivo, but all animals cleared the infection and recovered spontaneously within 48 h despite being almost depleted of neutrophils (<100/μL) [19].

### Development of the lethal model of pneumonia

Table 2 shows that addition of mucin to the optimized inoculum of *S. pneumoniae* INS-E611 for nasal instillation resulted in continuous growth of bacteria; the nadir occurred at 14 h (5.35 ± 0.53 log_10_ CFU/g) and the zenith at 38 h (9.26 ± 0.19 log_10_ CFU/g). The net growth during the 24 h period spanning from nadir to zenith (G_14→38h_) was 3.91 log_10_ CFU/g, and mice mortality reached 100% within the same period. Control mice instilled with mucin without bacteria remained healthy and had sterile lungs at 38 h.

**Table 2:** **Impact of inoculating bacteria without or with 5% porcine mucin in the model of pneumonia in neutropenic mice with diverse strains of penicillin non susceptible** ***Streptococcus pneumoniae*** **(PNSSP)**

	**Strain of Penicillin Non Susceptible** ***Streptococcus pneumoniae***													
**Parameter**	**ATCC 49619**		**INS-E611**		**INS-E674**		INS-E676*		INS-E678*		**INS-E683**		**INS-E684**	
**Inoculum preparation: Todd Hewitt broth (THB) without or with mucin**	**THB**	**Mucin + THB**	**THB**	**Mucin + THB**	**THB**	**Mucin + THB**	**THB**	**Mucin + THB**	**THB**	**Mucin + THB**	**THB**	**Mucin + THB**	**THB**	**Mucin + THB**
**Inoculum (mean, log**_**10**_ **CFU/mouse)**	7.12	6.34	6.95	6.78	6.46	6.79	6.28	6.20	6.53	6.53	6.71	6.61	7.00	6.25
**Time to nadir (h)**	14	14	14	14	14	14	**	**	**	**	14	14	14	14
**Time to zenith (h)**	42	38	36	38	48	38	***	***	***	***	36	38	48	38
**Bacterial count at nadir (mean log**_**10**_ **CFU/mouse ± SD)**	5.82 ± 0.09	6.32 ± 0.52	5.43 ± 0.39	5.35 ± 0.53	6.52 ± 0.65	7.34 ± 0.43	4.30 ± 0.36	3.97 ± 0.43	4.93 ± 0.06	6.02 ± 0.13	4.90 ± 0.17	5.70 ± 0.39	7.01 ± 0.72	6.73 ± 0.74
**Bacterial count at zenith (mean log**_**10**_ **CFU/mouse ± SD)**	8.02 ± 0.05	6.79 ± 0.44	7.58 ± 0.35	9.26 ± 0.19	8.70 ± 0.51	9.15 ± 0.34	5.09 ± 0.56	7.51 ± 0.18	6.68 ± 0.04	8.67 ± 0.28	7.77 ± 0.53	8.91 ± 0.75	9.59 ± 0.53	9.01 ± 0.52
**Growth from nadir to zenith (mean)**	2.19	0.47	2.15	3.91	2.18	1.63	---	---	---	---	2.87	3.21	2.58	2.28
**Growth from hour 14 to 38 (mean)**	1.24	0.47	−0.43	3.91	1.89	1.63	0.79	3.54	1.75	2.65	1.83	3.21	1.94	2.28
**Mouse Lethality (%)**	0	100	0	100	67	100	0	100	0	100	0	100	100	100
**Time to death (mean, h)**	All alive	64	All alive	52	56	52	All alive	44	All alive	44	All alive	59	61	51
**Bacterial count after death from pneumonia and sepsis (mean ± SD)**	All alive	8.21 ± 0.51	All alive	8.37 ± 0.39	9.52 ± 0.01	9.00 ± 0.30	All alive	7.89 ± 0.43	All alive	8.92 ± 0.05	All alive	8.52 ± 0.62	8.22 ± 0.38	8.76 ± 0.04

*All PNSSP strains were used in at least in two separate experiments, except for E676 and E678, which were tested once.

**Time fixed at 14 h.

***Time fixed at 38 h.

The addition of mucin to the bacterial inoculum transformed the pneumonia model from non-lethal (0%) to uniformly lethal (100%), and it correlated with a statistically significant increase in the bacterial burden of the lungs 38 h after infection (6.50 ± 0.43 without mucin vs. 9.26 ± 0.19 log_10_ CFU/g with mucin; P = 0.04 by Mann–Whitney test).

### Impact of neutrophils on the model

**Figure 2:**
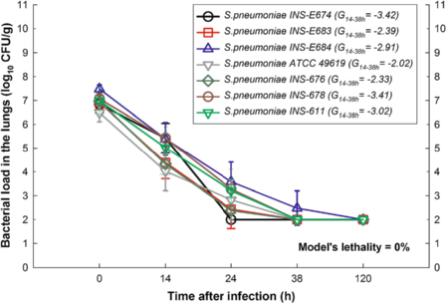
**Impact of neutrophils on the model.** In vivo growth dynamics of seven strains of PNSSP using an optimized inoculum in immune competent mice (normal neutrophil count). The animals exhibited no clinical signs of disease and cleared the pneumococci completely, indicating that neutropenia is a necessary requirement for a successful infection model. The open symbols represent the mean (at least three mice per time-point) and the error bars the standard deviation. The net bacterial growth between the 14 h and 38 h (G_14-38h_) was included into the legend box for each strain.

Fully immunocompetent mice spontaneously cleared the infection 120 hours after inoculation with any of the seven strains despite using the optimized inoculum supplemented with mucin (Figure 2). It shows that neutropenia is essential for the development of a successful model with PNSSP strains.

### Pulmonary histopathology

**Figure 3:**
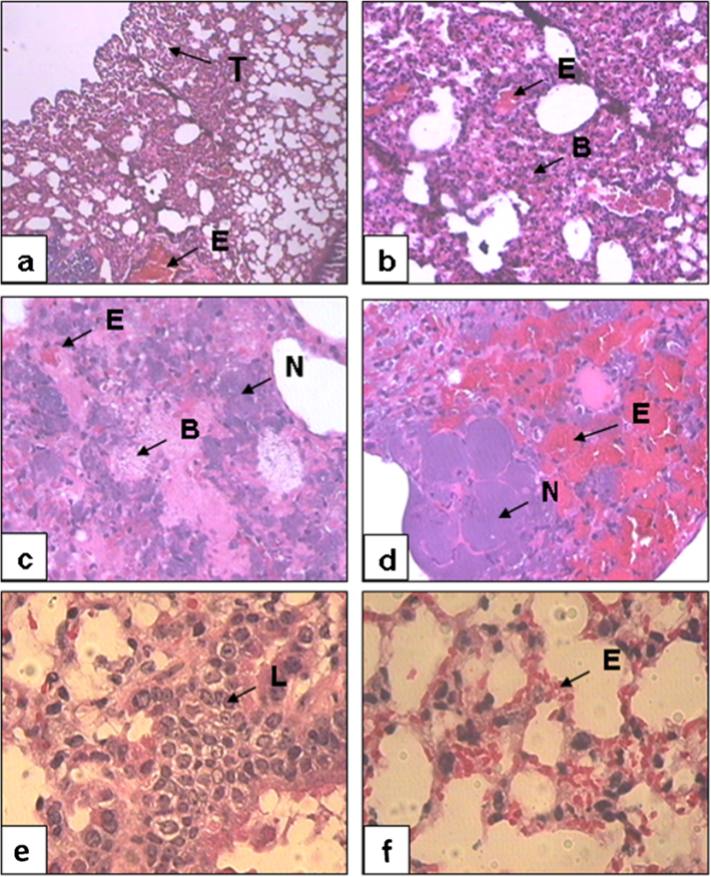
**Histopathological finding in lungs of mice infected with** ***S. pneumoniae*** **(with or without mucin) or instilled with sterile mucin.** Lung biopsies stained with hematoxylin-eosin and observed under optic microscopy with magnification of x4 (panels **a** and **c**), x10 (panels **b** and **d**) and x40 (panels **e** and **f**). Panels a and b correspond to mice infected with an inoculum of *S. pneumoniae* INS-E611 grown in Todd-Hewitt broth (THB) without mucin, panels c and d correspond to animals infected with mucin-supplemented inoculum and panels e and f show the lungs from uninfected mice instilled with sterile mucin. Abbreviations: necrosis (N), atelectasis (T), Gram positive bacteria (B), edema (E) and lymphocytes (L).

Figure 3 shows the histological findings in the lungs of the different groups of granulocytopenic mice sacrificed 38 h post-infection with *S. pneumoniae* INS-E611. Without mucin, mild changes were observed related to lymphocytic interstitial and hemorrhagic pneumonitis with some areas of atelectasis (panels a and b). In sharp contrast, the addition of mucin to the inoculum led to severe lung damage characterized by extensive septum edema, necrosis, and destruction of the alveolar structure; lymphocyte and mononuclear cell infiltrate and abundant bacteria accumulated within fibrin clots near alveolar septa (panels c and d). The expected polymorphonuclear infiltrate and subsequent lung consolidation of human pneumonia is not evident in this model because profound granulocytopenia was induced in the animals with cyclophosphamide [19].

Control mice nasally instilled with sterile broth and mucin only showed slight histological changes compatible with aspirational chemical pneumonitis without alveolar hemorrhage (non-specific lymphocytic interstitial pneumonitis). These animals never exhibited clinical signs of disease (Figure 3, panels e and f).

### Reproducibility of the lethal pneumonia model using optimized cultures with mucin of different PNSSP strains

**Figure 4:**
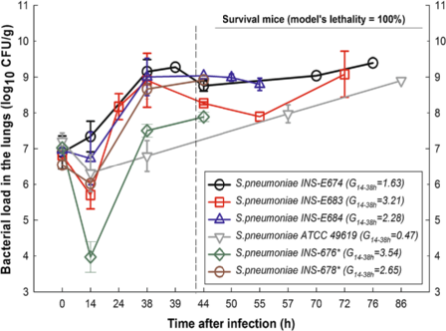
**Inter-strain reproducibility of pneumonia model using optimized culture condition with mucin.** In vivo growth dynamics of diverse strains of penicillin-non susceptible *S. pneumoniae* (INS-E674, E683, E684 and ATCC 49619) using an early log-phase inoculum supplemented with mucin (data from at least two different experiments). INS-676 and 678 (marked with an asterisk in the legend) were tested in one single experiment. The vertical dotted line indicates the time when a group of animals was sacrificed for bacterial counting in the lungs. Data after the line was obtained from animals left for survival assessment (all dead by the end of the experiment).

Table 2 summarizes the in vivo impact of inoculating bacteria without or with 5% porcine mucin. Clearly, a lethal pneumonia model with active bacterial growth in lungs was accurately established with all PNSSP strains tested (E611, E674, E676, E678, E683, E684, and ATCC 49619) after the addition of mucin to the bacterial inoculum, independently of their serotype or intrinsic murine virulence [17]. Besides, pneumococcal growth in the lungs was steadier with mucin and mortality reached 100% between 42 to 86 h after infection due to very high bacterial loads achieved in the lungs by the end of the model (Figure 4). Without mucin (despite identically optimized culture conditions), there was a wide variation in the time required to reach the zenith (36 to 48 h after inoculation) and only 2 of the 7 strains (E674 and E684) were lethal. All mice inoculated with 50 μL of sterile THB with mucin were healthy during 10 days of follow-up.

## Discussion

Animal models are indispensable tools for the study of infectious diseases and represent a link between *in vitro* and *in vivo* situations. In this regard, the possible translation of the results to humans demands the proof of “predictive validity”, meaning that reliability (which involves repeatability and reproducibility) and relevance (accuracy to predict the biological response) were determined during the standardization of the animal model [22]. However, respiratory tract infection models in mice using penicillin-resistant pneumococci strains from human infections has been a challenge. In fact, there is no evidence of predictive validity in the models used since 1980. According to our in vivo data without mucin (Figure 1), the high variability (SD > 1.3 log_10_ CFU/g at 48 h) on the bacterial load in lungs hampers the repeatability and reproducibility of the non-optimized model. Our findings with the non-optimized model are similar to those reported by Beskid et al. [23] and Azoulay-Dupuis et. al. [24], some of the most cited authors in this field.

Other pneumonia models by *S. pneumoniae* are available in the literature characterizing bacterial and host factors of virulence or testing the efficacy of antimicrobials and vaccines [25]. In general, these models require strains with capsular serotypes 2–6 because the other serotypes lack murine virulence [9,21].

Here, we improved the mouse pneumonia model by optimization of in vitro culture conditions and addition of mucin to the nasal inoculum, establishing a uniformly lethal infection. Additionally, the model was characterized by very high and steady bacterial counts in the lungs that correlated with histopathological signs of infection, even with strains belonging to serotypes 9V, 14, and 19F, known for their low virulence against *Mus musculus*.

Recently, we demonstrated the relevance of the pneumonia model in neutropenic mice to predict the biologic response [26]. Since the host’s immune system enhances significantly the efficacy of most antimicrobials, its elimination is necessary to determine the intrinsic bactericidal activity in vivo [19,27].

Nungester et al. pioneered the use of mucin to induce lethality to immunocompetent rats infected by intra-tracheal instillation of a fully susceptible (serotype 3) pneumococcus strain [12,28]. Using survival as outcome and detailed histopathology, they described extensively the role of mucin to enhance mortality. Our results demonstrate that optimization of in vitro culture conditions, mucin supplementation of the inoculum, and neutropenia are required to induce progressive growth of PNSSP in the lungs, and that success is achieved independently of the intrinsic virulence of the strain, its capsular serotype or resistance to penicillin. Although capsular type and antibiotic susceptibility are factors that determine the virulence of pneumococci [16,21,29,30], our optimized inoculum prevented their interference with the growth of pneumococci in vivo. Moreover, our method induces a degree of in vivo replication of *S. pneumoniae* that exceeds significantly the mean bacterial burden reported by other authors [14,31], with the additional advantages of low variability and short duration of the model (38 h). These characteristics are essential to ensure the reliability and relevance of the model and indispensable to prevent unnecessary suffering to the animals [22].

Regarding the use of mucin, all epithelial surfaces are covered and protected by mucus. Mucins are a family of large molecular weight glycoproteins with a high content of clustered oligosaccharides with O-glycosidic links to tandem repeat peptides rich in threonine, serine, and proline; additionally, they are major constituents of the mucus layer [32]. There are two distinct classes of human mucin, the secreted gel-forming (MUC2, MUC5AC, MUC5B, and MUC7) and the membrane-associated mucins, also called MAMs (MUC1, MUC3A, MUC3B, MUC4, MUC11, MUC12, MUC16) [33,34]. Each one of the human mucins has a related mucin in mice (named Muc instead of MUC to differentiate between species) [34]. Secreted mucins are produced by the goblet cells and serve to cover, hydrate and sweep away trapped foreign material from the uppermost coating of the epithelium. The MAMs are anchored to the apical epithelium cell membrane by single transmembrane domains, and serve as a glycocalyx barrier that prevents microbial adherence to airway epithelium. Most organs synthesize more than one type of mucin, although a specific type may predominate in a particular organ [32]. Previously, Adler et al. described that cell-free filtrates from broth cultures of *Pseudomonas aeruginosa*, *Haemophilus influenzae* and *Streptococcus pneumoniae* stimulate secretion of glycoconjugates by explants of guinea pig trachea [35]. In fact, they found that the extracellular product of *S. pneumoniae* that stimulates mucin secretion is a protein with a molecular weight ranging from 100,000 to 300,000 Da, and concluded that “the bacteria themselves may contribute to local manifestations” [35]. Here, we used one of the secreted mucins (purified porcin gastric mucin, pPGM type III, Sigma) to mimic the first action of the virulent strains exposed to the airway epithelia, that is, to secrete an extracellular protein with stimulatory effects on mucin production. Although the serine, threonine and proline repeat sequences of each MUC gene are species-specific, the cys-rich regions can be compared between different species to identify some similarity [36]. Thus, porcine gastric mucin (PGM), one of the most characterized mucins, has two fully sequenced clones (PGM-2A and PGM-9B) with function and structure related to the human mucins. Moreover, the arrangement of PGM-2A is identical with that reported for human intestinal mucin gene MUC2, while PGM-9B is related with MUC5AC [37,38].

Mucin supplementation works even for pneumococcal serotypes lacking murine virulence; the data suggest that mucin turns these strains lethal. This situation mirrors the clinical scenario in which viral infections like influenza predispose to secondary bacterial superinfections [39,40]. During the inflammatory response to viral infection, gene expression is upregulated for many molecules including the secreted mucins. Hypersecretion of mucin, increased viscoelasticity of mucus and decreased ciliary function in patients with viral respiratory infections or chronic diseases like asthma, COPD, and cystic fibrosis, can lead to airway obstruction and promote persistence of trapped pathogens in the airways [41]. In addition, epithelial mucins interact with several other respiratory pathogens including *Pseudomonas aeruginosa*, *Staphylococcus aureus*, *Haemophilus influenzae* and *Streptococcus pneumoniae* [42]. Bound pathogens persist in the airway and may initiate an inflammatory response mediated by virulence enhancement factors regulated by mucin, like the neuraminidase A [43].

Optimization of in vitro culture conditions was relevant for the success of the pneumococcal model. Recently, similar findings with enterococci [44] and coagulase-negative staphylococci have been reported [45]. The protocol specifying the optimal culture conditions required to obtain large numbers of young and healthy PNSSP cells in the early phase of growth without untimely activation of autolysis is already published [17]. Although little attention has been given to the role of in vitro conditions for bacterial growth in recent reviews of pneumococcal pneumonia models [5,10], scant bacterial growth (<1 log_10_ CFU/g) with huge variance (SD > 1 log_10_ CFU/g) are common characteristics of classic models of pneumococcal pneumonia [6,16,46,47]. Furthermore, models using non-optimized inocula of pneumococci in late-growth phase are proposed as protocols [25]. The methods and the data exposed here provide a simple mouse model of pneumococcal pneumonia with markedly improved reliability and relevance to determine the pharmacodynamics of antibiotics [15].

## Conclusions

Optimization of culture and inoculum conditions of PNSSP allows the induction of lethal pneumonia after nasal instillation of diverse strains to neutropenic mice, independently of the pneumococcal murine virulence. This model is suitable, reliable and reproducible for anti-infective pharmacology research.
